# Branding foods as ‘healthy’ or ‘unhealthy’ based on marginal data calls findings into question

**DOI:** 10.1007/s00125-022-05767-6

**Published:** 2022-08-16

**Authors:** Mitch Kanter

**Affiliations:** Alliance for Potato Research and Education, Chicago, IL USA

**Keywords:** Carbohydrates, Plant-based, Plant-forward, Potatoes, Type 2 diabetes

*To the Editor*: This letter is in response to the recently published manuscript entitled ‘Plasma metabolite profiles related to plant-based diets and the risk of type 2 diabetes’ by Hu and colleagues [1]. This manuscript assesses dietary factors that influence type 2 diabetes; given the astounding increase in diabetes prevalence in our society, the timeliness and gravity of this topic cannot be overstated. The authors conclude that plant-based diets are associated with a lower risk of developing diabetes, findings that complement a growing body of literature supporting this concept [1]. However, significant flaws in the study design and interpretation of selected results are cause for concern.

First, by distinguishing between ‘healthy’ and ‘unhealthy’ plant-based foods, the authors risk overstating the health effects of any given individual food item. The study identifies 264 metabolites, with 93 associated with the healthy plant index and 75 associated with the unhealthy plant index, yet only 50 and 32 metabolites, respectively, were unique to one group or the other [1]. This finding suggests a great deal of overlap in metabolites between the two groups. Noteworthy overlap exists between individual foods as well. For example, the metabolite profiles shown in Fig. 3 for nuts (in the ‘healthy plant foods’ group) and potatoes (in the ‘unhealthy plant foods’ group) are quite similar [1], which calls into question why one food is considered healthy while the other is considered unhealthy. Categorising potatoes as unhealthy is also questionable in light of US Department of Agriculture and Dietary Guidelines for Americans recommendations, which include three healthy food patterns characterised by regular consumption of starchy vegetables such as potatoes and refined grains [2]

Further, the reference cited to justify the grouping classification is a paper by Hu and colleagues from 2016 [3]. The authors of this study created a ‘healthy plant-based’ diet that excluded potatoes from the ‘healthy’ pattern, purportedly based on data from a publication by Martínez-González and colleagues [4]. Interestingly, Martínez-González and colleagues categorised potatoes as a ‘healthy plant-based’ food, and only limited evidence is provided in the current study by Hu and colleagues to rationalise the reassignment of potatoes to the ‘unhealthy plant foods’ category. More specifically, the rationale for this decision was based on a single 15-year-old observational study in female health providers that showed only modest associations between potato and French fry consumption and risk for type 2 diabetes. The decision by Hu and colleagues to re-classify potatoes as an unhealthy food based on such limited evidence is questionable.

Undoubtedly, lifestyle interventions are necessary to reduce the global burden of disease, especially regarding type 2 diabetes. Dietary modifications are a realistic and promising intervention strategy for not only improving human health, but also influencing the trajectory of our food system in favour of a plant-forward future. Nutrition researchers are responsible for producing objective data to guide dietary recommendations; unfortunately, the study in question falls short because of the characterisation of some individual foods as healthy or unhealthy based on seemingly subjective perspectives that are not supported by the totality of the available literature.

To this effect, carefully designed studies with objectively interpreted results are necessary to positively improve human health and shift agribusiness, nutrition policy and beyond in favour of a more sustainable, plant-forward food system.
